# Synthesis of KH550-Modified Hexagonal Boron Nitride Nanofillers for Improving Thermal Conductivity of Epoxy Nanocomposites

**DOI:** 10.3390/polym15061415

**Published:** 2023-03-13

**Authors:** Bolin Tang, Miao Cao, Yaru Yang, Jipeng Guan, Yongbo Yao, Jie Yi, Jun Dong, Tianle Wang, Luxiang Wang

**Affiliations:** 1 State Key Laboratory of Chemistry and Utilization of Carbon Based Energy Resources, College of Chemistry, Xinjiang University, Urumqi 830017, China; 2 Nanotechnology Research Institute, School of Materials and Textile Engineering, Jiaxing University, Jiaxing 314001, China; 3 Zhejiang Provincial Key Laboratory for Cutting Tools, School of Materials Science and Engineering, Taizhou University, Taizhou 318000, China; 4 College of Chemical and Materials Engineering, Zhejiang A&F University, Hangzhou 311300, China

**Keywords:** hexagonal boron nitride, epoxy, surface modification, polymer matrix nanocomposites, thermal conductivity

## Abstract

In this work, KH550 (γ-aminopropyl triethoxy silane)-modified hexagonal boron nitride (BN) nanofillers were synthesized through a one-step ball-milling route. Results show that the KH550-modified BN nanofillers synthesized by one-step ball-milling (BM@KH550-BN) exhibit excellent dispersion stability and a high yield of BN nanosheets. Using BM@KH550-BN as fillers for epoxy resin, the thermal conductivity of epoxy nanocomposites increased by 195.7% at 10 wt%, compared to neat epoxy resin. Simultaneously, the storage modulus and glass transition temperature (Tg) of the BM@KH550-BN/epoxy nanocomposite at 10 wt% also increased by 35.6% and 12.4 °C, respectively. The data calculated from the dynamical mechanical analysis show that the BM@KH550-BN nanofillers have a better filler effectiveness and a higher volume fraction of constrained region. The morphology of the fracture surface of the epoxy nanocomposites indicate that the BM@KH550-BN presents a uniform distribution in the epoxy matrix even at 10 wt%. This work guides the convenient preparation of high thermally conductive BN nanofillers, presenting a great application potential in the field of thermally conductive epoxy nanocomposites, which will promote the development of electronic packaging materials.

## 1. Introduction

The rapid development of modern electronic devices toward high integration, high power density, and miniaturization presents an increasing requirement for heat dissipation, which makes it urgent to develop high thermally conductive materials for thermal management applications [1,2,3,4]. Currently, the most commonly used thermal management materials (TMMs) are mainly some types of thermosetting polymers, such as epoxy resin [5], organosilicone [6], polyurethane [7,8], etc. Among them, epoxy resin receives extensive research as a TMM, by virtue of its high adhesion strength, chemical durability, and excellent mechanical strength [9,10,11]. However, the low thermal conductivity of epoxy resins (~0.2 W m^−1^K^−1^) is far from meeting the need for efficient heat dissipation of modern electronic devices. Therefore, developing TMMs with high thermal conductivity is of great importance for the electronic and electrical industry.

Incorporating high-thermal-conductivity filler into epoxy matrix is a widely used method for improving the thermal conductivity of epoxy resin [12,13]. Typical thermal conductive fillers include metals (Ag, Al, etc.) [14], carbon-based materials (carbon nanotube, graphene, etc.) [15,16,17], and ceramics (Al_2_O_3_, BN, etc.) [18,19]. Compared with metals or carbon-based materials, ceramic fillers usually exhibit good electrical insulating characteristic besides high thermal conductivity, which is quite suitable for use as thermally conductive fillers of TMMs. As a ceramic filler with a similar two-dimensional (2D) structure of graphene, hexagonal BN has excellent thermal conductivity and electrical insulating property, especially the BN nanosheets exfoliated from BN that possess a theoretical thermal conductivity as high as ~2000 W m^−1^K^−1^ [20,21]. Nevertheless, the strong atom interactions between neighboring planar B and N atoms, commonly referred to as “lip-lip” interactions, make exfoliating BN significantly more difficult [22].

To date, different means have been explored for the preparation of BN nanosheets, such as mechanical ball-milling [23], chemical vapor deposition [24], and thermal exfoliation [25]. However, these exfoliation methods still face three main challenges to practical application: the low yield (Y) of BN nanosheets (<20%), difficult separation of BN nanosheets, and poor interfacial compatibility between BN nanosheets and polymer matrix. Usually, the obtained BN nanosheets need to be further surface-modified with silane coupling agents or other organic molecules in the application of polymer composites [26].

**Scheme 1:**
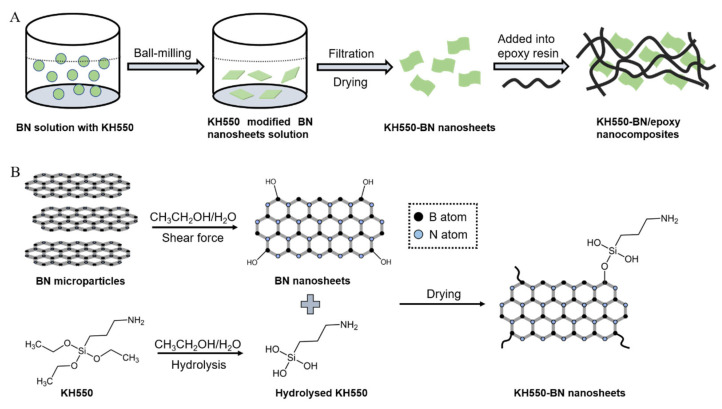
Illustration for preparation of BN/epoxy nanocomposites (**A**) and modification process of BN fillers (**B**).

Recently, Chen et al. [27] developed a simple and efficient one-step method for the preparation of functionalized few-layer BNNPs by solid-state ball milling of commercially available h-BN and urea powder. Ren et al. [28] reported that the yield and dispersibility of BN nanosheets can be effectively enhanced via a tannic acid (TA)-assisted liquid-phase exfoliation, due to the intermolecular interaction. Among these methods, the interaction between organic molecules and BN, such as Lewis acid–base interactions, was considered to play a critical role in the exfoliation of BN. Moreover, Agrawal’s group [29] and Liu’s group [11] demonstrated that by using aminosilane-coupling-agent-modified BN as fillers, the thermal conductivity of BN/epoxy composites can be improved effectively. Herein, the KH550-modified BN nanopowders with high content of nanosheets were synthesized via a one-step ball-milling process for improving the thermal conductivity and mechanical property of epoxy nanocomposites. As shown in Scheme 1, the BN particles were directly added into a mixed solution of deionized water (H_2_O) and absolute ethyl alcohol (CH_3_CH_2_OH) containing KH550, followed by performing the ball-milling process. On one hand, the -NH_2_ group (lewis base) of KH550 can interact with B atom (Lewis acid) of BN; on the other hand, the mechanical impact and shear forces generated by grinding balls could make KH550 molecules easy to insert the gap of BN layers. As a result, a high yield of BN nanosheets was obtained, and the KH550 were also grafted onto the surface of BN nanosheets under the action of ball-milling internal heat. Using ball-milled KH550-modified BN as fillers, epoxy nanocomposites were prepared after blending and curing, and the thermal conductivity and dynamic thermomechanical property of epoxy nanocomposites were investigated.

## 2. Materials and Methods

### 2.1. Materials

BN powders (hexagonal, 99.9%, ~3 μm), γ-aminopropyl triethoxy silane (KH550, 97%), and absolute ethyl alcohol (99.7%) were purchased from McLean Biochemical Technology Co., Ltd. (Shanghai, China). Epoxy resin (E-51), methylhexahydrophthalic anhydride (MHHPA), and 2.4.6-Tri(dimethylaminomethyl)Phenol (DMP-30) were provided by Haining Hailong Chemical Co., Ltd. (Jiaxing, China). All raw materials or reagents were not further purified before use.

### 2.2. Preparation of BN Nanofillers

Raw BN (R-BN) powders were added into a stainless steel ball grinding tank containing 50 stainless steel grinding balls, followed by pouring a mixed solution of H_2_O and CH_3_CH_2_OH (volume ratio: 3:7) containing 1% KH550 into the ball-grinding tank at a ratio of 0.05 g·mL^−1^ [23]. Subsequently, the ball-grinding tanks were loaded onto the planetary ball mill, and the ball-milling process was performed at a speed of 500 r/min for 8 h at room temperature. After this, the BN powders in mixed solution were collected by vacuum filtration, and washed three times with deionized water to remove the unreacted KH550 molecules. After they were dried for 12 h at 60 °C in a vacuum-drying oven, the KH550-modified BN nanofillers synthesized by one-step ball-milling (BM@KH550-BN) were obtained. As a contrast, another BN nanofiller, denoted as BM-BN, was prepared according to the same procedure as BM@KH550-BN, except no KH550 was used in the mixed solution of H_2_O and CH_3_CH_2_OH. In addition, the BM-BN were also post-modified with 1% KH550 solution at 60 °C for 2 h to obtain the conventional KH550-modified BN nanofillers (BM-KH550-BN).

### 2.3. Preparation of BN/Epoxy Nanocomposites

The desired ratios of BN nanofillers were ultrasonically dispersed into absolute ethyl alcohol at a concentration of 0.05 g·mL^−1^, and then the epoxy resin was added into the BN dispersion. With vigorous stirring, the mixed solution of BN/epoxy was reduced-pressure distilled at 60 °C until the absolute ethyl alcohol was fully evaporated. Afterwards, the MHHPA and DMP-30 were added into the mixed solution in sequence, followed by stirring for 30 min at room temperature in a vacuum. Finally, the BN/epoxy nanocomposites were obtained after BN/epoxy mixture was cured at 120 °C for 2 h in stainless steel mold. Herein, the weight ratio of epoxy resin, MHHPA, and DMP-30 was set to 100:85:0.8, and the weight content of BN nanofillers was set to 1%, 4%, 7%, and 10%.

### 2.4. Characterization and Measurement

The micromorphology of BN nanofillers and BN/epoxy nanocomposites were investigated by field-emission scanning electron microscope (FE-SEM, Apero 2, a resolution of 1 nm, Thermo Scientific, Waltham, USA) equipped with an energy-dispersive spectrum (EDS) component and transmission electron microscope (TEM, Talos F200X, Thermo Scientific, Waltham, MA, USA). The samples for EDS were prepared by natural deposition BN nanofillers on a polished Ti substrate. The fracture surfaces of the epoxy nanocomposites were prepared by cryo fractured in liquid nitrogen. The optical photographs of BN nanofillers solution (2 mg·mL^−1^) were taken on a white paper by a smartphone (Mate 40, Huawei, Shenzhen, China). Fourier-transform infrared (FTIR) spectra of BN nanofillers were recorded at room temperature by an infrared spectrometer (Bruker, Vertex 70) with the range from 400 cm^−1^ to 4000 cm^−1^. The thermogravimetric analysis (TGA) was performed by a thermal gravimetric analyzer (TA Instruments (New Castle, DE, USA), Q50) at a heating rate of 5 °C/min from 30 to 800 °C under a nitrogen atmosphere according to the ASTM E1131 standard. Thermal conductivity of BN/epoxy nanocomposites (50 mm in diameter and 20 mm in height) was tested with a quick thermal conductivity meter (Xiangyi instrument, DRE-III). The dynamic thermomechanical properties of BN/epoxy nanocomposites were measured with DMA 850 (TA Instruments) according to the ASTM standard D4065-94. The nanocomposite samples were cut into strip-shaped specimens (60 × 10 × 4 mm^3^) to match with machine. A temperature scan was conducted from 50 to 200 °C with a heating rate of 5 °C/min at a frequency of 1 Hz and a strain of 0.05% in a double cantilever mode. The storage modulus and tan δ (the ratio of loss modulus to storage modulus) were obtained from DMA analysis, and the glass transition temperature (Tg) was gained from the peak value of tan δ.

## 3. Results and Discussion

### 3.1. Morphology and Element Mapping of BN Nanofillers

**Figure 1:**
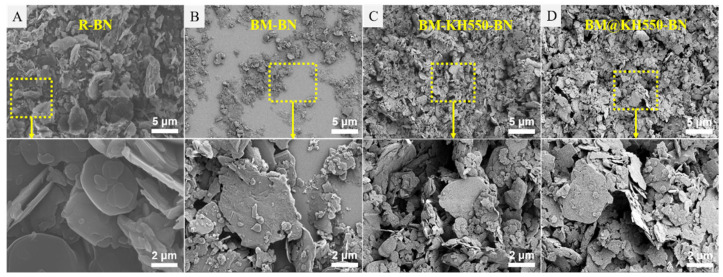
SEM images of different BN nanofillers (**A**–**D**).

Using raw BN (R-BN) powders as materials, three different kinds of BN nanofillers were prepared by ball-milling method, i.e., BM-BN, BM-KH550-BN, and BM@KH550-BN. As shown in Figure 1A, the R-BN particles present the typical stacked layered structure and uneven sizes, with an approximate size range from 1 to 8 μm. After ball-milling treatment with an aqueous solution (Figure 1B), the obtained BN particles seem to have a smaller size and thinner thickness than R-BN, and some BN nanosheets appear in BM-BN. This can be attributed to the impact and shear effect of the ball-milling process [30]. It is also found that the post-modification with KH550 coupling agents does not change the morphology of BM-BN nanofillers (Figure 1C). When aqueous solution is substituted with KH550 solution in the ball-milling process, a large number of BN nanosheets are observed in the obtained BM@KH550-BN nanofillers (Figure 1D), suggesting the successful exfoliation of R-BN particles in the KH550-assisted ball-milling process.

**Figure 2:**
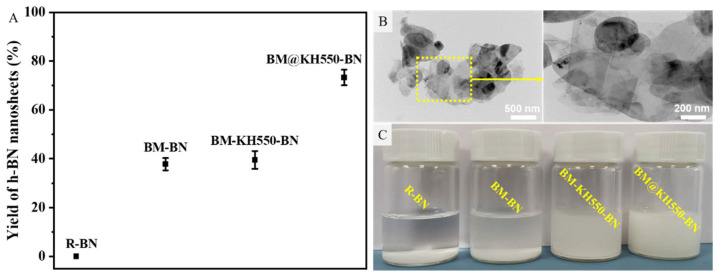
The yield of BN nanosheets in different BN nanofillers (**A**); TEM images of BM@KH550-BN nanofillers (**B**); optical photographs of different BN nanofillers ethanol dispersion after standing for 24 h (**C**).

To estimate the ratio of BN nanosheets (exfoliated BN) in different nanofillers, a centrifugal separation method was performed at 3000 rpm. Results indicate that the BM-BN and BM-KH550-BN exhibit a similar yield of BN nanosheets (~38%), indicating that the surface modification with KH550 does not change the amount of BN nanosheets. However, a yield of BN nanosheets as high as 73.3% is gained in BM@KH550-BN nanofillers (Figure 2A). This may be ascribed to the fact that the electron donor -NH_2_ in KH550 can readily insert into the layered structure of BN with the assistance of mechanical force and weaken the atom interactions between electron acceptor B atom and neighboring planar N atom, resulting in the higher yield of BN nanosheets [31]. For the BM@KH550-BN nanofillers, the unambiguous BN nanosheets structure are observed in TEM images (Figure 2B). The dispersibility of BN nanofillers were further evaluated by observing the stability of BN ethanol dispersion. It can be seen from Figure 2C that the R-BN dispersion almost becomes clear, and BM-BN dispersion is a little turbid after standing for 24 h. It is noteworthy that the BM-KH550-BN and BM@KH550-BN dispersion are still relatively turbid after 24 h, especially BM@KH550-BN, which is ascribed to the fact that the KH550 silane coupling agent can improve the dispersibility of nanofillers, and, simultaneously, the high ratio of BN nanosheets in BM@KH550-BN is also conducive to the dispersibility of nanofillers.

**Figure 3:**
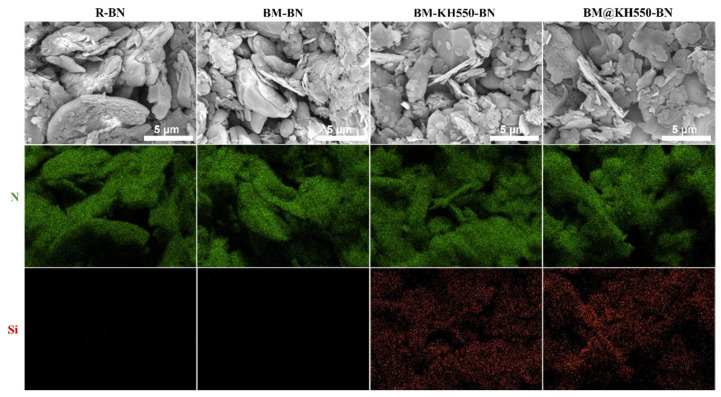
Nitrogen (N) and silicon (Si) element mapping images of different BN nanofillers.

The key element distribution of different BN nanofillers were detected by EDS (BN nanofillers deposited on polished Ti substrate). The element mapping images in Figure 3 show that all the BN nanofillers exhibit a strong nitrogen (N) element signal (green) with uniform distribution. For the silicon (Si) element (red), it is found that no Si element signal is detected in R-BN and BM-BN samples. However, the apparent Si element signals are observed in the BM-KH550-BN and BM@KH550-BN nanofillers, which implies that the KH550 molecules are successfully bound to the surface of BN nanofillers during the ball-milling process, since the Si element only exists in KH550 molecules.

### 3.2. FTIR Analysis of BN Nanofillers

**Figure 4:**
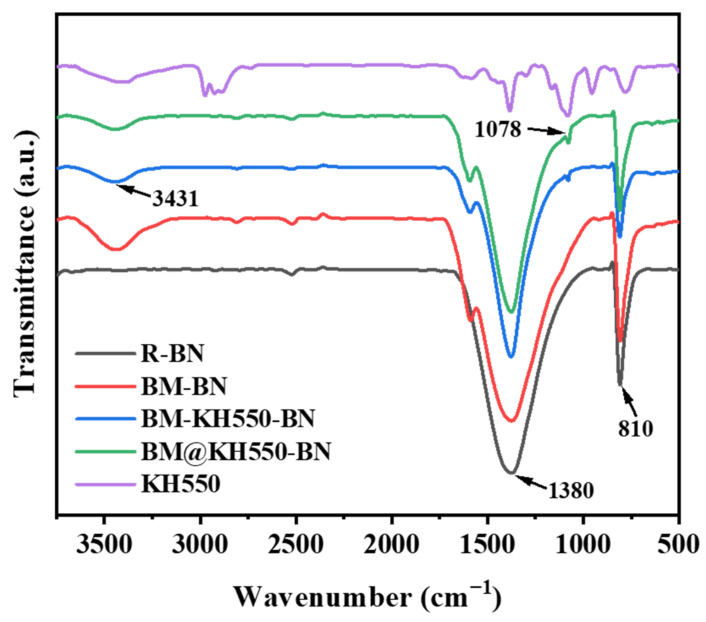
FTIR spectra of different BN nanofillers.

In order to further investigate the chemical compositions of BN nanofillers, the FTIR spectra of BN nanofillers were recorded at room temperature. As shown in Figure 4, the two strong adsorption bands at 1380 and 810 cm^−1^ are indexed to the in-plane stretching vibration and out-of-plane bending vibration of B-N bond, respectively [32,33]. It is found that the adsorption band of typical -OH stretching vibration at 3431 cm^−1^ is hardly found in R-BN, but an obvious adsorption band is observed in ball-milling-treated BN nanofillers (BM-BN, BM-KH550-BN, and BM@KH550-BN), which indicates that the ball-milling treatment enhances the degree of hydroxylation of R-BN [34]. It is noteworthy that a new adsorption band at 1078 cm^−1^, which is indexed to the stretching vibration of the Si-O bond of KH550, emerges in BM-KH550-BN and BM@KH550-BN, implying that KH550 is successfully grafted onto the surface of BN nanofillers through the ball-milling process [35].

### 3.3. TGA Analysis of BN Nanofillers

**Figure 5:**
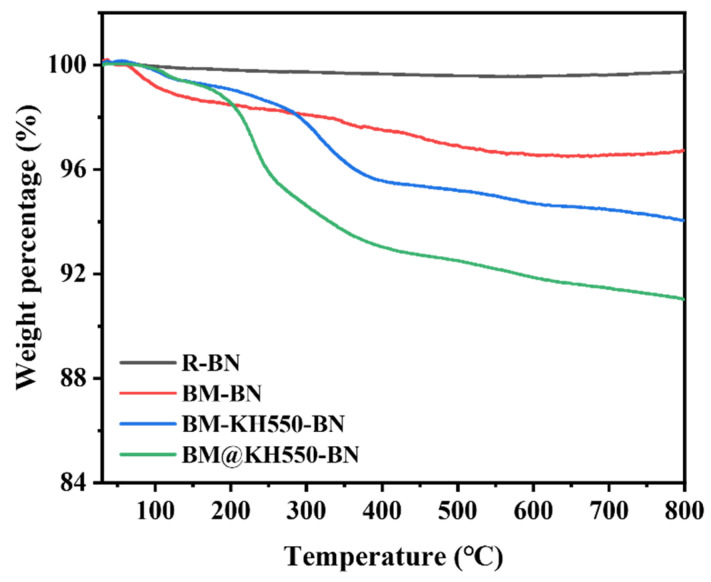
TGA curves of different BN nanofillers.

The thermal stability of BN nanofillers was analyzed by TGA. It can be seen from Figure 5 that the R-BN shows little weight loss (only 0.25%) over the whole temperature range, but the weight loss of BM-BN is estimated to 3.26%, which is mainly ascribed to the removal of adsorbed water and -OH groups on the surface of BM-BN [36]. In addition, the weight loss of BM-KH550-BN and BM@KH550-BN are determined to 5.96% and 9.97%, respectively, implying the higher content of KH550 molecules on the BN surface. The weight loss of BM-KH550-BN and BM@KH550-BN is mainly attributed to the removal of adsorbed water and -OH groups, and the decomposition of KH550 molecules [37]. The weight loss of all the BN nanofillers mainly takes place between 100 °C and 400 °C. It is noteworthy that the initial weight loss (below 200 °C) of BM-BN is larger than other BM-KH550-BN and BM@KH550-BN, which may be due to the higher level of -OH groups, as confirmed by FTIR spectra. Compared to BM-KH550-BN, the higher content of KH550 in BM@KH550-BN usually indicates the better interfacial compatibility between nanofillers and epoxy resin.

### 3.4. Thermal Conductivity Analysis of BN Nanofillers

**Figure 6:**
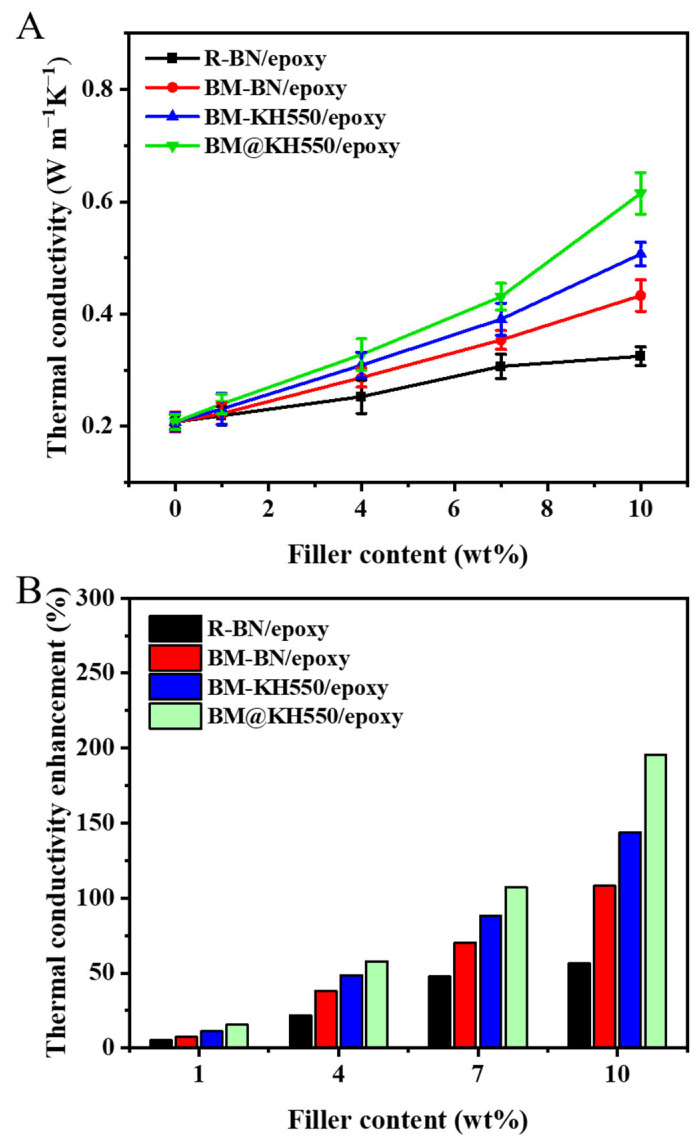
Thermal conductivity (**A**) and thermal conductivity enhancement (**B**) of different BN/epoxy nanocomposites.

Using the as-prepared BN nanoparticles as fillers, the BN/epoxy nanocomposites with different content of BN nanofillers were prepared, and the thermal conductivity property of BN/epoxy nanocomposites was measured by thermal conductivity meter. The testing result in Figure 6A shows that the thermal conductivity of the neat epoxy is only 0.208 W m^−1^K^−1^, which is attributed to the fact that the highly cross-linked network structure of epoxy resin presents a low crystallinity and orderliness, which is not enough to make phonons propagate quickly [38]. When the four different BN nanofillers are added into epoxy matrix, the thermal conductivity of epoxy resin has an insignificant enhancement at 1 wt%. Although the weight content of BN nanofillers increases to 4%, the highest thermal conductivity with a value of 0.328 W m^−1^K^−1^ is obtained in the BM@KH550/epoxy nanocomposite, which is only 57.7% higher than neat epoxy. This can be understood by the fact that the small amount of thermally conductive BN nanofillers make it difficult to form an effective heat conduction path inside the epoxy resin. After the content of BN nanofillers is greater than 4%, the thermal conductivity of all the BN/epoxy nanocomposites enhances significantly with the increase in BN nanofillers, due to the gradual contact with each other among BN nanofillers. It is found that the thermal conductivity of R-BN/epoxy, BM-BN/epoxy, BM-KH550/epoxy, and BM@KH550/epoxy nanocomposites at 7 wt% increase to 0.307, 0.354, 0.391, and 0.431 W m^−1^K^−1^, respectively. When the content of BN nanofillers is further increased to 10 wt%, the enhancement of thermal conductivity of four epoxy nanocomposites presents relatively large differences. For R-BN/epoxy nanocomposites, the thermal conductivity is only enhanced to 0.325 W m^−1^K^−1^ from 0.307 W m^−1^K^−1^, which may be ascribed to the fact that the poor dispersion of R-BN nanofillers and the worse interfacial compatibility between R-BN nanofillers and epoxy matrix produces a lot of R-BN aggregation formations inside the R-BN/epoxy nanocomposite, hindering the conduction of heat. However, the BM-BN/epoxy, BM-KH550/epoxy, and BM@KH550/epoxy nanocomposites are determined to be 0.433, 0.507, and 0.615 W m^−1^K^−1^, which is increased by 108.2%, 143.8%, and 195.7%, respectively (Figure 6B), compared to the thermal conductivity of neat epoxy resin. The highest thermal conductivity in BM@KH550/epoxy nanocomposites may be ascribed to the good interfacial compatibility between the BM@KH550-BN nanofillers and epoxy matrix and the high content of BN nanosheets in BM@KH550-BN nanofillers, forming more efficient thermal conductive paths in BM@KH550/epoxy nanocomposite.

### 3.5. Thermomechanical Properties of the Epoxy Nanocomposites

**Figure 7:**
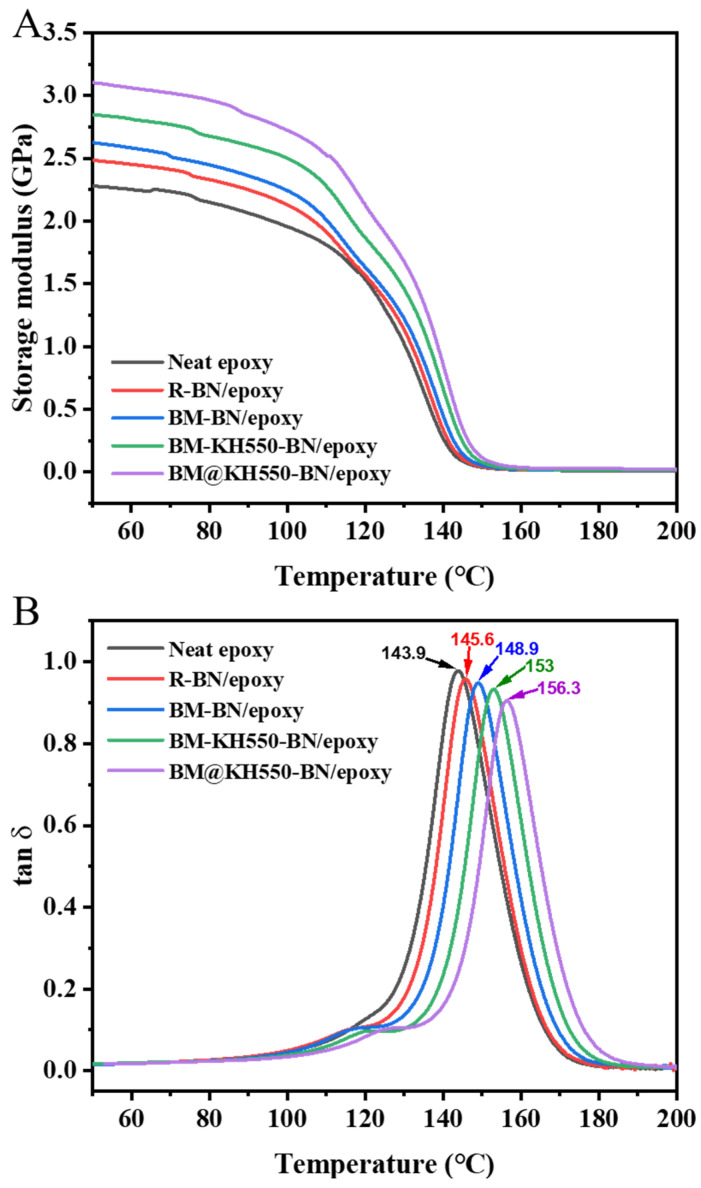
Temperature-dependent storage modulus (**A**) and tan δ (**B**) curves of different BN/epoxy nanocomposites at 10 wt%.

The dynamic thermomechanical properties of BN/epoxy nanocomposites were further tested. The test results show that the storage modulus of neat epoxy resin is 2.28 GPa (Figure 7A). After incorporation of the 10 wt% BN nanofillers, the storage modulus of neat epoxy resin enhances in different degrees. For R-BN/epoxy, the storage modulus only increases from 2.28 GPa to 2.49 GPa, mainly ascribed to the easy aggregations of raw BN particles at high content. For BM-BN/epoxy, a storage modulus of 2.63 GPa is achieved, due to the existence of some BN nanosheets in the BM-BN nanofillers. It is found that the storage moduli of BM-KH550/epoxy and BM@KH550/epoxy nanocomposites at 10 wt% reaches 2.85 and 3.09 GPa at 50 °C, respectively, which is 25% and 35.6% higher than that of neat epoxy resin, respectively. This is mainly ascribed to the fact that the reactive amino groups on the surface of the BM-KH550 and BM@KH550 nanofillers enhance the interfacial bonding strength between nanofillers and epoxy matrix, and the surface of BM@KH550 has more abundant reactive amino groups [39]. In addition, the glass transition temperature (Tg) of all the epoxy nanocomposites at 10 wt% were analyzed by the peak value of tan δ curves. As shown in Figure 7B, the Tg of neat epoxy resin is determined to be 143.9 °C, and an insignificant enhancement in Tg is found in the R-BN/epoxy (145.6 °C). For the BM-BN/epoxy and BM-KH550-BN/epoxy nanocomposites, the Tg is determined to be 148.9 °C and 153 °C, respectively, which is higher than the Tg of neat epoxy. Expectedly, the BM@KH550-BN/epoxy nanocomposite presents the highest Tg, with a value reaching up to 156.3 °C, which is 12.4 °C higher than that of neat epoxy resin. The enhanced Tg in the BM@KH550-BN/epoxy nanocomposite is mainly ascribed to the fact that the covalent connection between amino groups in BM@KH550-BN and epoxy groups in the epoxy molecule chain restrain the mobility of the local epoxy molecules around the BM@KH550-BN nanofillers, enhancing the degree of cross-linking [40]. It is worth noting that the height of tan δ peak reduces gradually from neat epoxy to the four kinds of epoxy nanocomposites (R-BN/epoxy, BM-BN/epoxy, BM-KH550/epoxy, and BM@KH550/epoxy nanocomposites), implying a decrease in the amount of mobile epoxy chains in epoxy nanocomposites [41].

### 3.6. Effectiveness of the BN Nanofillers and Constrained Region of the Epoxy Nanocomposites

To explore the effect of different BN nanofillers on the dynamic thermomechanical properties of epoxy matrix, the effectiveness of BN nanofillers and constrained region of the epoxy nanocomposites were analyzed. The factor β_f_ represents the effectiveness of fillers on the moduli of composites, and can be given by the equation:

(1) $$\beta_{f}=\frac{({G_{g}}^{\prime }/{G_{r}}^{\prime })\;\mathrm{composite}}{({G_{g}}^{\prime }/{G_{r}}^{\prime })\;\mathrm{matrix}}$$

where G_g_′ and G_r_′ are the storage modulus in the glassy region and rubbery region, respectively [42]. The G_g_′, G_r_′, and β_f_ of epoxy nanocomposites are listed in Table 1. The lower β_f_ value represents the higher effectiveness of the filler. Clearly, all the epoxy nanocomposites have a lower β_f_ value than neat epoxy. Also, the BM@KH550-BN/epoxy nanocomposite exhibits the lowest β_f_ value (79.2) among the four types of BN nanofillers (R-BN/epoxy, BM-BN/epoxy, BM-KH550/epoxy, and BM@KH550/epoxy nanocomposites), indicating the strongest reinforcing effect of BM@KH550 nanofillers on the epoxy matrix. In addition, the incorporation of fillers in polymer matrix can also affect the entanglement dynamics and mobility of the polymer chains around the fillers. Herein, the volume fraction of the constrained region can be quantitatively calculated by the tan δ value. The relationship between tan δ and energy loss fraction (W) of the polymer nanocomposites is given by the equation [43]:

(2) $$W=\frac{\pi \;\tan\;\delta }{\pi \;\tan\;\delta +1}$$

where the energy loss fraction W at the tan δ peak can be expressed by the dynamic viscoelastic data with the following equation:

(3) $$W=\frac{\left(1-C\right){\;W}_{0}}{1-C_{0}}$$

where C is the volume fraction of the constrained region of the composite, and W_0_ and C_0_ are the energy loss fraction and volume fraction of the constrained region of neat epoxy, respectively [44]. Equation (3) can be rearranged as follows:

(4) $$C=1-\frac{\left(1-C_{0}\right)\;W}{W_{0}}$$

Herein, C_0_ is taken as zero for neat epoxy, and the C value of epoxy nanocomposites are calculated by Equation (4). As listed in Table 1, the volume fraction of the constrained region in the R-BN/epoxy nanocomposite is only 0.004, indicating the weak effect of R-BN on the epoxy chains. However, the volume fraction of the constrained region in the BM-BN/epoxy and BM-KH550/epoxy is higher than that of the R-BN/epoxy nanocomposite, implying the stronger effect of BM-BN and BM-KH550 nanofillers on the epoxy chains than R-BN nanofillers. For BM@KH550-BN/epoxy nanocomposite at 10 wt%, the volume fraction of the constrained region reaches 0.02, which is higher than that of the other epoxy nanocomposites. This may be explained by the fact that a stronger interfacial attraction exists between BM@KH550-BN nanofillers and epoxy matrix, better restricting the mobility of the epoxy chains [45].

### 3.7. Fracture Surface Analysis of the Epoxy Nanocomposites

**Figure 8:**
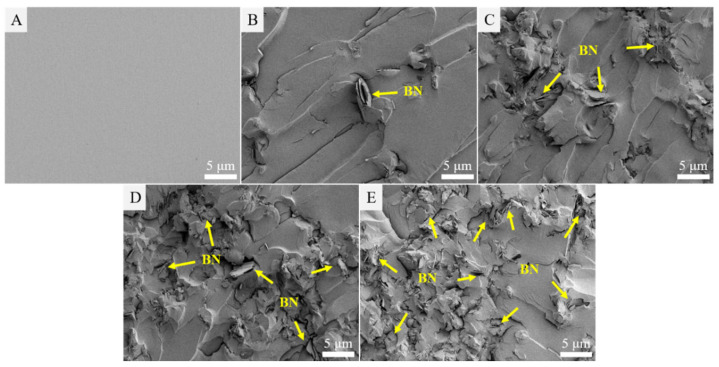
The SEM images of fracture surface of neat epoxy (**A**) and BM@KH550-BN/epoxy nanocomposites with different content: 1% (**B**), 4% (**C**), 7% (**D**), 10% (**E**).

As is well known, the distribution of fillers in polymer matrix plays an important role in the thermal conductivity and thermomechanical properties of epoxy nanocomposites. Since the BM@KH550-BN/epoxy nanocomposite achieves the optimal thermal conductivity and thermomechanical properties in all the epoxy nanocomposites, it is necessary to investigate the distribution of BM@KH550-BN nanofillers at different loading content in epoxy matrix. Thus, the morphology of fracture surfaces of BM@KH550-BN/epoxy nanocomposites was analyzed. As shown in Figure 8A, the neat epoxy displays a flat and clean fracture surface, which is a typical characteristic of epoxy resin [46,47]. After the incorporation of 1% BM@KH550-BN nanofillers into the epoxy matrix, an uneven fracture surface and little BN particles (yellow arrow) embedded in epoxy matrix are observed, and the small area of irregular cracks and slightly rougher fracture surface for the composites are also observed (Figure 8B). It is found that the unevenness of the fracture surface of the epoxy nanocomposite and the amount of BN particles increase obviously with the increase in BM@KH550-BN nanofillers, and the large area of irregular cracks and the roughness of the fracture surface is also enhanced greatly (Figure 8C–E). When the weight content of BM@KH550-BN nanofillers reaches 10 wt%, a large number of BN particles emerge on the fracture surface of the epoxy nanocomposite. It should be noted that the BM@KH550-BN nanofillers still display a uniform distribution in the epoxy matrix even if the content of BM@KH550-BN is 10%, which indicates the good dispersion of BM¬@KH550-BN nanofillers in epoxy matrix.

## 4. Conclusions

In summary, the KH550-modified BN (BM@KH550-BN) nanofillers were successfully prepared via a one-step ball-milling route, and a high proportion of BN nanosheets and excellent dispersion stability were confirmed in BM@KH550-BN nanofillers. As a result, the BM@KH550-BN nanofillers can enhance the thermal conductivity of epoxy nanocomposites significantly, with an enhancement of 195.7% at 10 wt%, compared to neat epoxy resin. In addition, the storage modulus and glass transition temperature (Tg) of BM@KH550-BN/epoxy nanocomposites also increase by 35.6% and 12.4 °C, respectively. The calculated results from the dynamical mechanical analysis show that the BM@KH550-BN nanofillers have the strongest reinforcing effect and the higher volume fraction of constrained region in all the epoxy nanocomposites. This is mainly attributed to the high ratio of BN nanosheets in BM@KH550-BN nanofillers and the good interfacial compatibility between nanofillers and matrix. This work develops a simple way to prepare high thermally conductive BN nanofillers, showing a good application prospect in the field of thermally conductive polymer nanocomposites.

## Figures and Tables

**Table 1:** Some characteristic parameters of epoxy nanocomposites at 10 wt% derived from DMA.

Samples	G_g_′ (GPa)	G_r_′ (GPa)	β_f_	W	C
Neat epoxy	2.28	0.011	207.3	0.754	0
R-BN/epoxy	2.49	0.013	191.5	0.751	0.004
BM-BN/epoxy	2.63	0.018	146.1	0.747	0.009
BM-KH550-BN/epoxy	2.85	0.026	109.6	0.746	0.011
BM@KH550-BN/epoxy	3.09	0.039	79.2	0.739	0.02

## Data Availability

The data presented in this study are available on request from the corresponding author.
